# Toxic risk of stereotactic body radiotherapy and concurrent helical tomotherapy followed by erlotinib for non-small-cell lung cancer treatment - case report

**DOI:** 10.1186/1471-2407-10-696

**Published:** 2010-12-31

**Authors:** Chen-Hsi Hsieh, Hou-Tai Chang, Shih-Chiang Lin, Yu-Jen Chen, Li-Ying Wang, Yen-Ping Hsieh, Chien-An Chen, Ngot-Swan Chong, Shoei Long Lin, Chun-Yi Chen, Pei-Wei Shueng

**Affiliations:** 1 Department of Radiation Oncology, Far Eastern Memorial Hospital, Taipei, Taiwan; 2 Department of Chest Medicine, Division of Internal Medicine, Far Eastern Memorial Hospital, Taipei, Taiwan; 3 Department of hematology, Far Eastern Memorial Hospital, Taipei, Taiwan; 4 Department of Radiation Oncology, National Defense Medical Center, Taipei, Taiwan; 5 Institutes of Traditional Medicine, School of Medicine, National Yang-Ming University, Taipei, Taiwan; 6 Department of Radiation Oncology, Mackay Memorial Hospital, Taipei, Taiwan; 7 School and Graduate Institute of Physical Therapy, College of Medicine, National Taiwan University, Taipei, Taiwan; 8 Department of Healthcare Administration, Asia University, Taichung, Taiwan; 9 Department of Surgery, Taipei Hospital, Department of Health, Taipei, Taiwan; 10 Division of Medical Oncology, Department of Internal Medicine, Taipei Hospital, Department of Health, Taipei, Taiwan

## Abstract

**Background:**

Stereotactic body radiation therapy (SBRT) applied by helical tomotherapy (HT) is feasible for lung cancer in clinical. Using SBRT concurrently with erlotinib for non-small cell lung cancer (NSCLC) is not reported previously.

**Case Presentation:**

A 77-year-old man with stage III NSCLC, received erlotinib 150 mg/day, combined with image-guided SBRT via HT. A total tumor dose of 54 Gy/9 fractions was delivered to the tumor bed. The tumor responded dramatically and the combined regimen was well tolerated. After concurrent erlotinib-SBRT, erlotinib was continued as maintenance therapy. The patient developed dyspnea three months after the combined therapy and radiation pneumonitis with interstitial lung disease was suspected.

**Conclusions:**

Combination SBRT, HT, and erlotinib therapy provided effective anti-tumor results. Nonetheless, the potential risks of enhanced adverse effects between radiation and erlotinib should be monitored closely, especially when SBRT is part of the regimen.

## Background

Erlotinib, one of the epidermal growth factor receptor (EGFR) tyrosine kinase inhibitors (TKIs), is active and relatively well tolerated in chemotherapy-naïve elderly patients with advanced non-small cell lung cancer (NSCLC) [1]. Image-guided stereotactic body radiotherapy (SBRT) and helical tomotherapy (HT) using hypofractionation in patients with early-stage medically inoperable NSCLC is feasible and well tolerated [2]. For stage III NSCLC, hypofractionation yields equivalent survival rates, but without often fatal symptomatic pneumonitis, compared to conventional radiotherapy [3]. The addition of standard-dose erlotinib to chemoradiotherapy is feasible, without an increase in toxicity [4]. Little information is available on fatal pulmonary toxicity due to irradiation pneumonitis when erlotinib is concurrently given with SBRT and used thereafter as maintenance therapy for NSCLC.

## Case presentation

**Figure 1:**
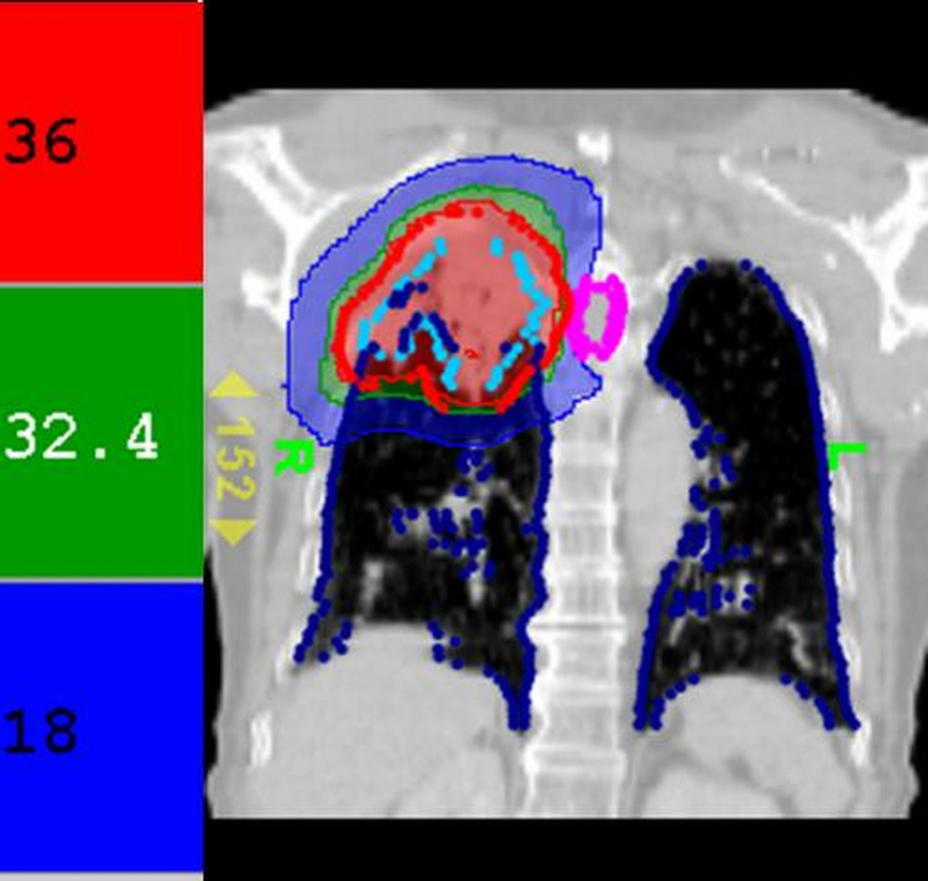
**Dose distribution in the first treatment course**. Tomotherapy treatment planning with high conformity (conformal index, CI = 1.03). Red, green, and blue areas are 100%, 90%, and 50% of the prescribed radiation dose, respectively. The blue dots outline the lung structure and the sky-blue dots indicate the radiation target.

**Figure 2:**
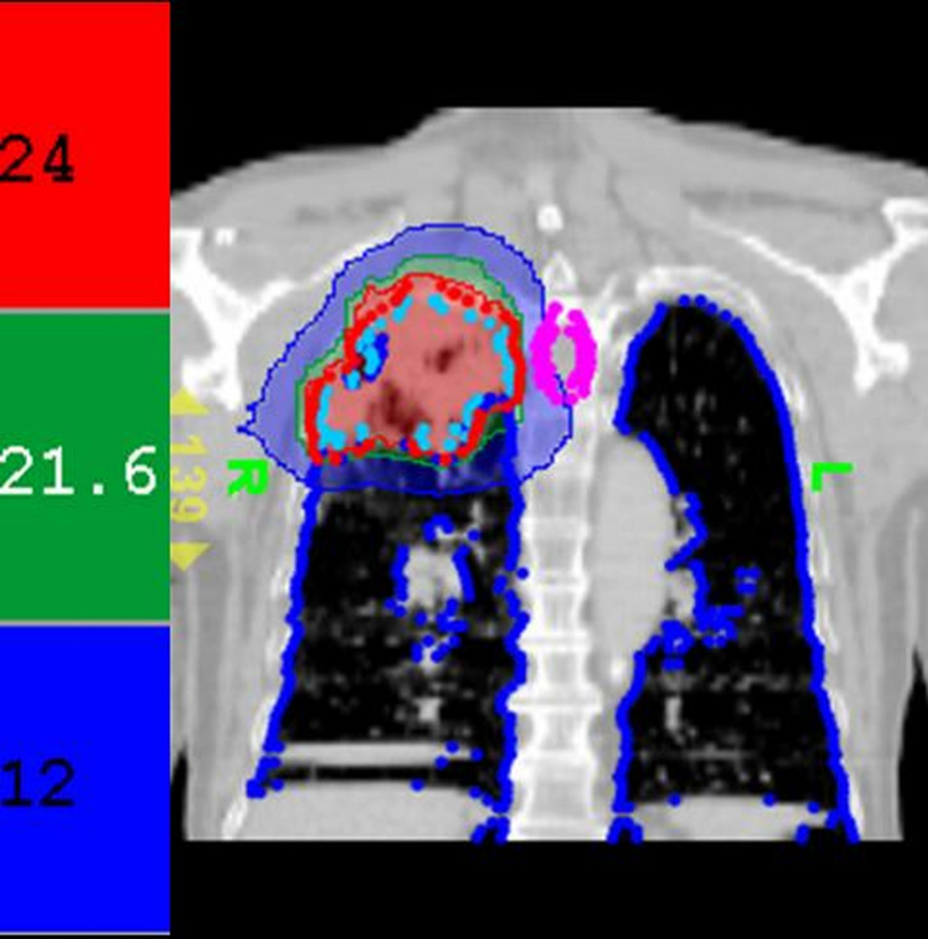
**Dose distribution in the second treatment course**. Tomotherapy treatment planning with high conformity (conformal index, CI = 1.03). Red, green, and blue areas are 100%, 90%, and 50% of the prescribed radiation dose, respectively. The blue dots outline the lung structure and the sky-blue dots indicate the radiation target.

A 77-year-old man was diagnosed with NSCLC, cT2N2M0, stage III A. Chest computed tomography (CT) showed a soft tissue mass measuring 4 × 3.9 cm in the right upper lung, with mediastinal lymphadenopathy. Carcinoembryonic antigen (CEA) was also elevated to 12.9 mg/dl. The Patient received oral erlotinib 150 mg/day as the first line therapy. Three months later, the CEA increased from 12.9 ng/ml to 29.1 ng/ml. Then, erlotinib was added concurrently to the radiotherapy regimen. This regimen comprised 54 Gy given in nine fractions delivered with SBRT using HT, at 95% of the prescribed isodose for the planned target volume. The split courses with 3 fractions per week were prescribed. (Figure 1 and 2) Targeting was based on new, separate CT scans for each split course.

**Figure 3:**
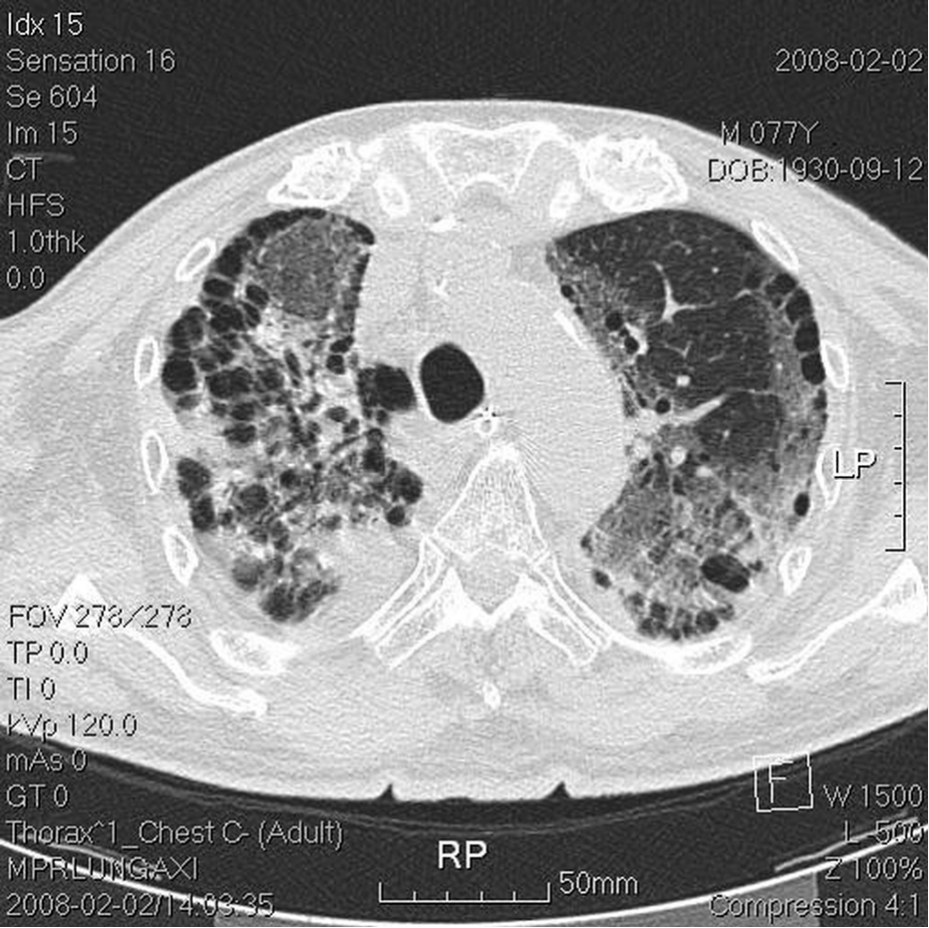
**Transverse view of upper lung field with tumor located in the right upper lung apex**. Chest computed tomography (CT) done post intubation shows ground-glass opacities, blebs confined to the right upper lung apex, and diffuse ground-glass attenuation, blebs in the marginal areas, airspace consolidation, and fibrosis in the bilateral upper and lower lung fields.

**Figure 4:**
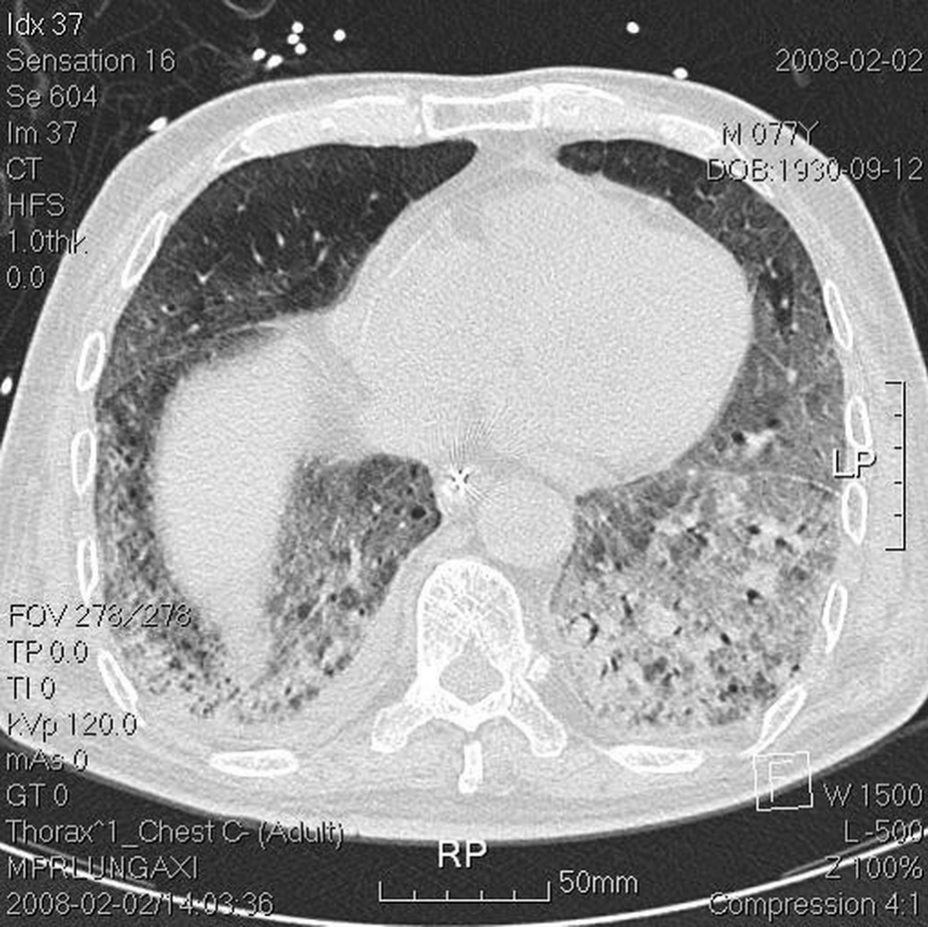
**Transverse view of the lower lung field**. Chest computed tomography (CT) done post intubation shows ground-glass opacities, blebs confined to the right upper lung apex, and diffuse ground-glass attenuation, blebs in the marginal areas, airspace consolidation, and fibrosis in the bilateral upper and lower lung fields.

**Figure 5:**
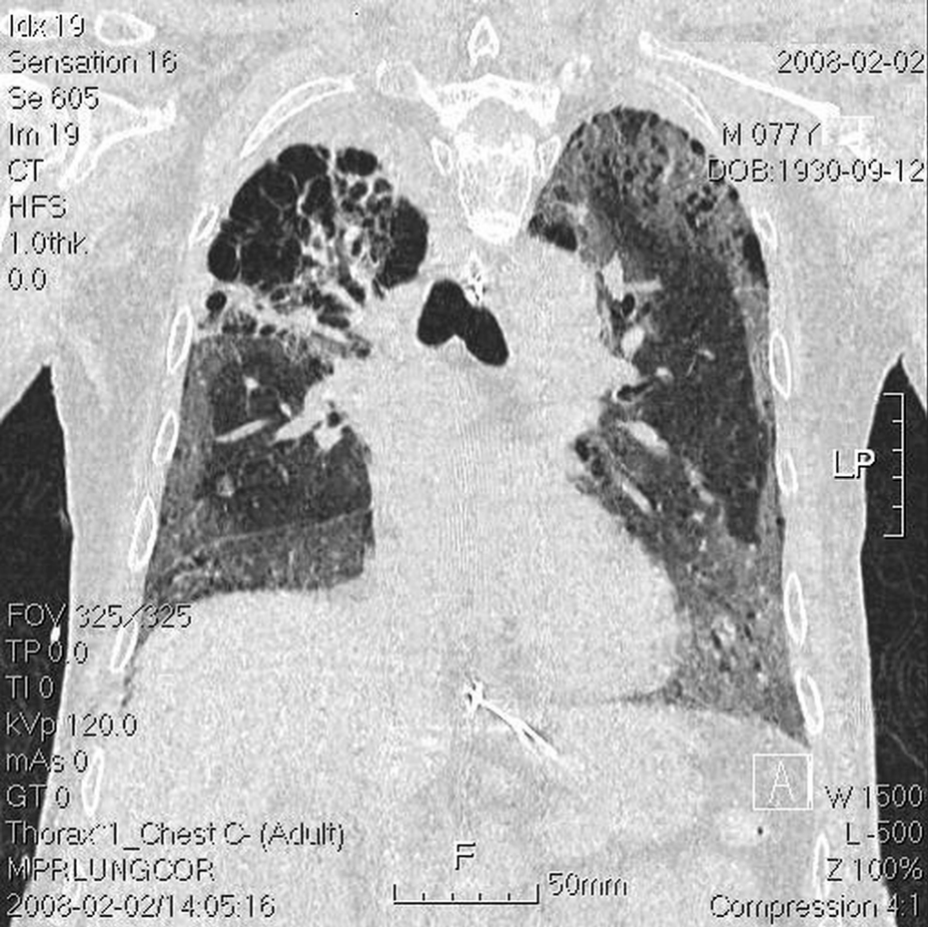
**Coronal view of the whole lung field**. Chest computed tomography (CT) done post intubation shows ground-glass opacities, blebs confined to the right upper lung apex, and diffuse ground-glass attenuation, blebs in the marginal areas, airspace consolidation, and fibrosis in the bilateral upper and lower lung fields.

**Figure 6:**
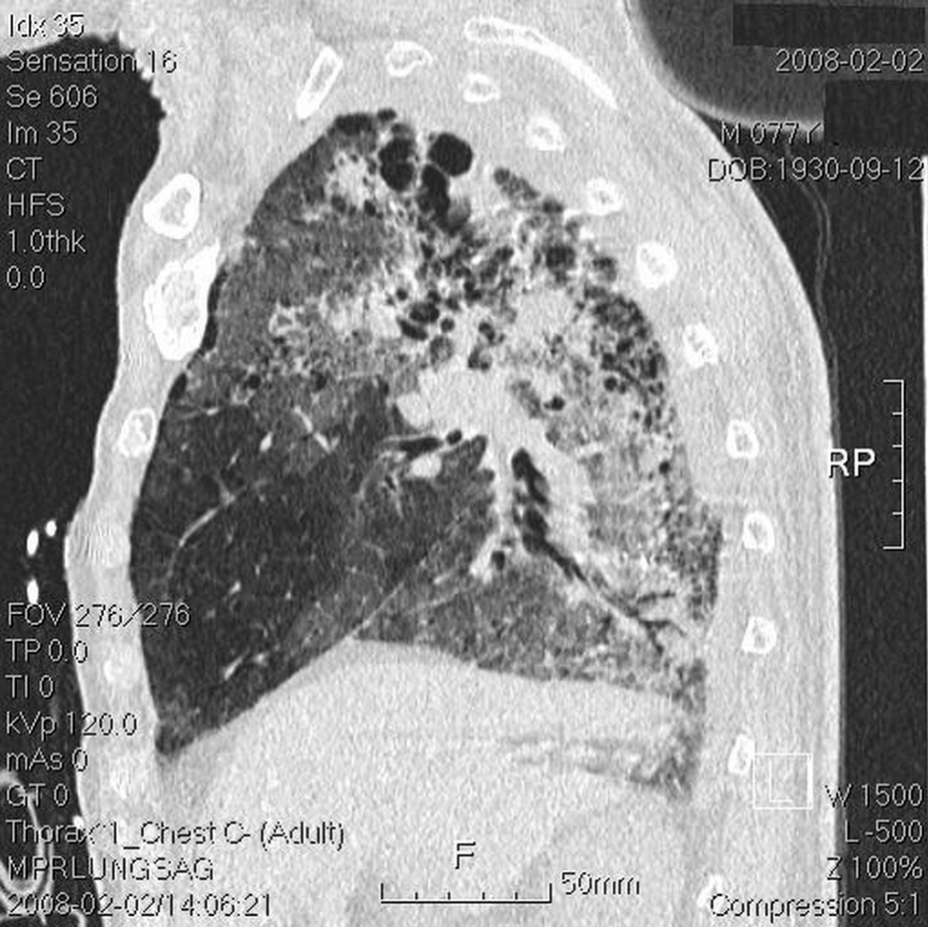
**Sagittal view of the whole lung field**. Chest computed tomography (CT) done post intubation shows ground-glass opacities, blebs confined to the right upper lung apex, and diffuse ground-glass attenuation, blebs in the marginal areas, airspace consolidation, and fibrosis in the bilateral upper and lower lung fields.

The tumor volume (ml) *vs. *the right lung volume was 116.1 ml *vs. *1282.9 ml in the first treatment course and 90.9 ml *vs. *1475.9 ml in the second treatment course. The mean lung dose, V15, and V20, where Vx was the percentage of lung volume that received at least × Gy [5] for separate lung images, is shown Table 1. The whole-course V20 and mean lung dose for the total lung were 10% and 10.24 Gy, respectively. By 2.5 months after the combination therapy, the tumor shrank from 4 × 3.9 × 4.5 cm to 2.4 × 2.9 × 2.1 cm and erlotinib 150 mg/day was prescribed as maintenance therapy. Unfortunately, the patient developed dyspnea three months after the combination therapy. He was transferred to the medical intensive care unit. In a series of image studies, opacities of a diffuse ground-glass pattern, subpleural bleb formation in the marginal areas, airspace consolidation and fibrosis in bilateral whole lung fields were noted, and radiation pneumonitis was suspected (Figure 3, 4, 5, 6) [6,7]. The patient received empirical antibiotics, steroid therapy, antioxidant, and supportive treatment. Four more months after the combined therapy, the patient died of respiratory failure.

**Table 1:** Mean lung volume, dose, V15, and V20 for each lung in the first and second radiotherapy courses, with 2 weeks interval between radiotherapy courses.

	First course (0-36 Gy)		Second course (36-54 Gy)	
	
	Right lung volume, 1167 ml	Left lung volume, 1244 ml	Right lung volume, 1385 ml	Left lung volume, 1490 ml
Mean lung dose (Gy)	10.77 Gy	4.74 Gy	4.63 Gy	1.96 Gy
V15* (%)	27%	2%	13%	0%
V20* (%)	22%	0%	8%	0%

*V15 and V20 are the percentages of lung volume that receive at least 15 and 20 Gy in the ipsilateral and contralateral lungs.

## Conclusions

Image-guided SBRT with HT using hypofractionation in patients with early-stage medically inoperable NSCLC is feasible [2]. The hypofractionated scheme yields equivalent survival rates, without fatal, symptomatic pneumonitis for patients with stage III NSCLC when compared with conventional radiotherapy [3]. Belderbos et al. [8] reported that radiation dose escalation was safe up to 94.5 Gy in 42 fractions with a mean lung dose 13.6 Gy or less in 6 weeks in NSCLC patients. The patients underwent irradiation 5 days per week, and twice daily when more than 30 fractions were prescribed, with at least a 6-h interval in between each fraction. According to linear-quadratic (LQ) modeling [9], the biologic effect of 94.5 Gy/42 fractions converted to a hypofractional dose of 6 Gy per fraction (EQD6), for which the acute effects and late normal tissue effects would be equivalent to 72 and 54 Gy, respectively. The mean lung dose (≥ 21 Gy), V20 (> 31%) [10], and ipsilateral V20 Gy [5] correlates with radiation pneumonitis. Nonetheless, the Radiation Therapy Oncology Group 0236 protocol using SBRT via HT for NSCLC provided safe and effective treatment when the V20 was restricted to less than 10% to 15% [11]. The V15, V20, and mean lung dose for each separate lung by divided course are shown in the Table. Moreover, the whole-course V20 and mean total lung dose were 10% and 10.24 Gy, respectively. According to previous reports [5,10,11], this plan was safe and no symptomatic radiation pneumonitis occurred among the NSCLC patients.

Erlotinib, an EGFR TKI, is an effective anti-tumor agent for treatment of NSCLC among elderly patients [1]. Erlotinib could be used as a single agent in select subsets of patients with advanced NSCLC [12]. In a comparative trial, only 0.8% of patients developed interstitial lung disease in the erlotinib arm [13]. Moreover, addition of standard-dose erlotinib to chemoradiotherapy was feasible and without evidence of increased toxicities [4]. However, prior tissue injury from radiation therapy could lead to cells with altered responses when the drug is subsequently applied [14]. Erlotinib enhanced radiation responses including cell cycle arrest, apoptosis induction, accelerated cellular repopulation, and DNA damage repair [15]. Therefore, it is possible for erlotinib to induce an altered response in cells when erlotinib is applied after irradiation.

Though SBRT applied by HT allows for minimization of normal tissue exposure to high radiation doses [16], the large amount of low-dose irradiation to non-target organs at risk (OAR), and thus, the incidence of lung toxicity can become high [17]. Recently, non-target OARs were impacted by arc therapy due to the low dose bath phenomenon and these effects could be magnified by agents known or unknown to be associated with recall effects [18]. Irradiation modulates the anticancer drug's pharmacokinetics even under low doses and in off-target areas [19]. Additionally, combined low-dose radiation and erlotinib induced symptomatic pneumonitis in one NSCLC patient [20]. Another NSCLC patient developed radiation recall dermatitis induced by erlotinib [21]. According to these reports, we believe EGFR inhibitor might not only enhance the effects of radiation, but also might enhance the adverse effects of radiation, especially when prescribed following previous concurrent treatment with radition. Furthermore, radiation modulates the systemic efficts of drugs regardless of the treatment effects or side effects. Erlotinib appears to modulate the effects of irradiation, both good and bad.

To our best knowledge, this is the first report of radiation pneumonitis caused by erlotinib combined with image-guided SBRT via HT with hypofractionation followed by erlotinib presecribed for maintenance. Oncologists should be alert to the potential risk of fatal pulmonary toxicity caused by this multimodality treatment. Radiotherapy plus targeting agents must be conducted in well-designed clinical trials.

## Consent

Written informed consent was obtained from the patient's family for publication of this case report and the accompanying images. A copy of the written consent is available for review by the Editor-in-Chief of the journal.

## Competing interests

The authors declare that they have no competing interests.

## Authors' contributions

CHH and PWS perfromed all CT evaluations, designed the study, made the target delineations, and interpreted the study results. CHH drafted the manuscript. SCL, CAC, SLL, CYC and HTC care for the patient. YJC, LYW, and YPH advised on all aspects of the work. NSC participated in radiation plannning. All authors read and approved the final manuscript.

## Pre-publication history

The pre-publication history for this paper can be accessed here:

http://www.biomedcentral.com/1471-2407/10/696/prepub
